# Supplementary material for: Increased H-Bond Stability Relates to Altered ε-Cleavage Efficiency and Aβ Levels in the I45T Familial Alzheimer’s Disease Mutant of APP
Source: Sci Rep. 2019 Mar 29;9:5321. doi: 10.1038/s41598-019-41766-1 (PMC6440955; doi:10.1038/s41598-019-41766-1)
Supplement: Supplementary file 1 — Supporting informations [file 41598_2019_41766_MOESM1_ESM.docx]

**SUPPLEMENTARY INFORMATION**

**Increased H-Bond Stability Relates to Altered ε-Cleavage Efficiency and Aβ Levels in the I45T Familial Alzheimer’s Disease Mutant of APP**

Alexander Götz^1§^, Philipp Högel^2§^, Mara Silber^3^, Iro Chaitoglou^2^, Burkhard Luy^3^, Claudia Muhle-Goll^3^, Christina Scharnagl^1*^ and Dieter Langosch^2*^

^1^Lehrstuhl für Physik synthetischer Biosysteme (E14), Technische Universität München, Maximus-von-Imhof Forum 4, 85354 Freising, Germany

^2^Center for Integrated Protein Science Munich (CIPSM) at the Lehrstuhl für Chemie der Biopolymere, Technische Universität München, Weihenstephaner Berg 3, 85354 Freising, Germany

^3^Institute of Organic Chemistry and Institute for Biological Interfaces 4, Karlsruhe Institute of Technology, Karlsruhe, Germany.

^§^These authors contributed equally.

^*^Corresponding authors

D. Langosch, Lehrstuhl für Chemie der Biopolymere, Technische Universität München, Weihenstephaner Berg 3, 85354 Freising, Germany. Tel.: +49-8161-71-3500; Fax: +49-8161-71-4404; Email: [langosch@tum.de](mailto:langosch@tum.de)

C. Scharnagl, Lehrstuhl für Physik synthetischer Biosysteme (E14), Technische Universität München, Maximus-von-Imhof Forum 4, 85354 Freising, Germany. Tel.: +49-8161-71-3557; Email: christina.scharnagl@tum.de

**Supplementary Discussion**

**The Strength of Amide Hydrogen Bonding within Transmembrane Helices: Amide D/H Exchange Kinetics vs. Fractionation Factors**

The strength of amide H-bonds within TMD helices in membrane mimics containing water has been characterized by measuring either (i) the kinetics of amide D-to-H exchange with H_2_O (alternatively: H-to-D exchange with D_2_O), or (ii) the equilibrium of D/H exchange in mixed H_2_O/D_2_O solutions.^1–6^ In order to investigate the connection between both approaches, we used the thermodynamic cycle shown in Supplementary Fig. S1.


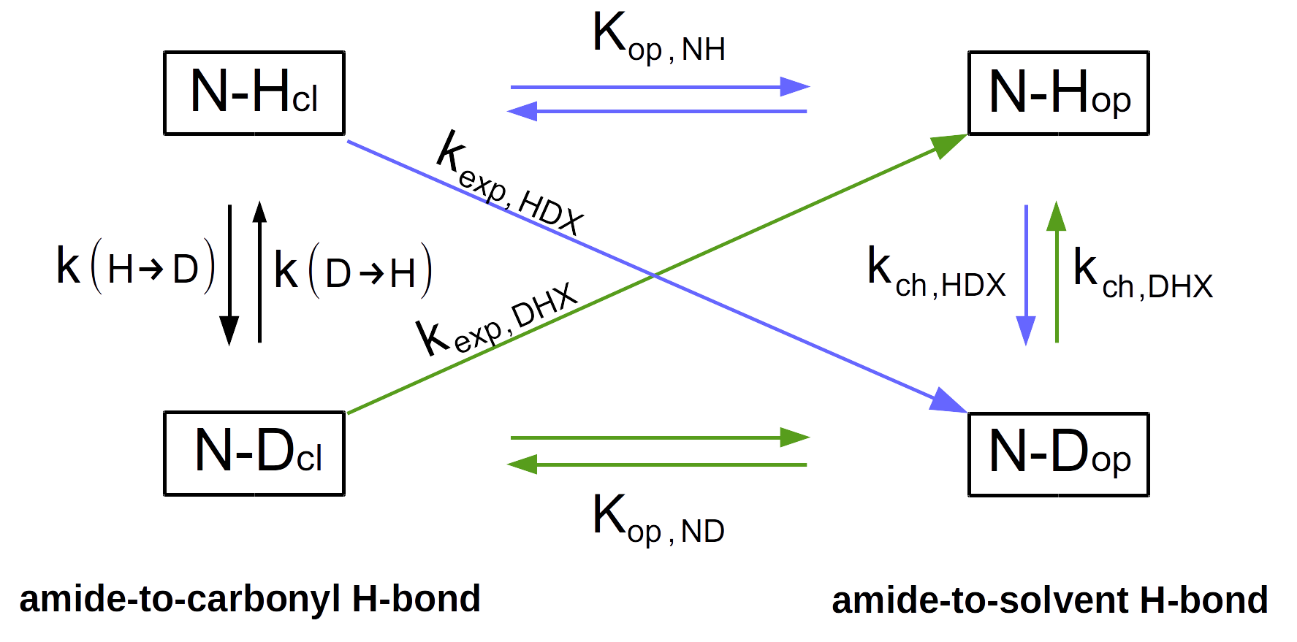


**Supplementary Figure S1:** Thermodynamic cycle describing amide isotope effects. In the 2-state model, a backbone amide either forms an intrahelical H-bond (“cl”, closed) or is exposed to the solvent after local unfolding (“op”, open). The populations of open and closed H-bonds determine the stability constants K_op,NH_ and K_op,ND_ for protiated and deuterated amides (open/close equilibria in upper and lower row). In a mixed H_2_O/D_2_O solvent, both classes of amide sites can enrich the isotope to a certain amount. When the exchange is at equilibrium, the isotopic preference at the amide relative to the concentration of that isotope in the aqueous solvent is measured by the fractionation factor Φ. The thermodynamic cycle connects the relative deuterium enrichment at an amide in the native state to its isotope preference in an unfolded, water-exposed state and to the relative open/close equilibria of protiated and deuterated H‑bonds in which the amide is the donor. The blue arrows indicate reactions contributing to H-to-D exchange kinetics from the protiated native state amide (N-H_cl_) in the EX2 regime, where a fast open/close equilibrium precedes slow exchange from the open state with the rate constant k_ch,HDX_. Reactions contributing to the D-to-H exchange kinetics are highlighted in green.

Under the conditions of our kinetic experiments (see Methods), D/H exchange follows the EX2 reaction scheme and is characterized by rate constants k_exp,HDX_ and k_exp,DHX_ (blue and green arrows in Supplementary Fig. S1**).** Here, fast open/close equilibria (K_op,NH_ and K_op,ND_) precede slow exchange with rate constants k_ch,HDX_ and k_ch,DHX_ from the small fraction of amides with a water‑exposed (open) H-bond:

|  | $k_{exp,DHX}\approx K_{op,ND}k_{ch,DHX}$ and $k_{exp,HDX}\approx K_{op,NH}k_{ch,HDX}$ | (1) |
| --- | --- | --- |

The stability of an intrahelical H-bond is characterized by the free energy ΔG_op_ of the open/close equilibrium and can be determined from D-to-H exchange kinetics according to the Linderstrøm-Lang theory^7–9^, e.g., for D-to-H exchange kinetics:

|  | $\Delta G_{op}=-RT\ln K_{op,ND}$ | (2) |
| --- | --- | --- |

A variety of experiments^4,10–13^ suggested previously that strong H-bonds tend to accumulate protium relative to its content in a mixed D_2_O/H_2_O solvent, while weak H-bonds accumulate deuterium. Therefore, the equilibrium constant Φ (the fractionation factor) of the isotope exchange of a backbone amide hydrogen with a solvent deuteron was suggested as a measure of the strength of an intrahelical amide H-bond (see Supplementary Fig. S1):

|  | $\Phi={\frac{[N-D_{cl}]}{[N-H_{cl}]}}/{\frac{[D_{2}O]}{[H_{2}O]}}$ | (3) |
| --- | --- | --- |

A fractionation factor Φ<1 reflects the enrichment of protons in strong H-bonds relative to the solvent, while a value of Φ=1 reflects the equal distribution of protons and deuterons between the amide group and the solvent.^10,12,14^ Generally, the isotope preference in an H-bond is governed by a complex interplay between (i) the stiffness of vibrational modes in which the N‑D or N-H covalent bond is involved, (ii) the geometry of the H-bond, and (iii) the pK_a_ values of donor and acceptor.^12,15^ However, the general relation of backbone amide Φ values to H‑bond stabilities or H-bond lengths is a controversial issue and even depends on individual interpretations of experiments.^10,11,16^

Isotope exchange in TM helices may be too slow to reach the equilibrium required to measure thermodynamic fractionation factors. Cao et. al.^5^ suggested to circumvent the problem of prohibitively slow exchange and calculated the native state’s fractionation factor from the kinetic isotope effect (see Supplementary Fig. S1),

|  | $\Phi={\frac{k(H\to D)}{k(D\to H)}}/{\frac{[D_{2}O]}{[H_{2}O]}}$ | (4) |
| --- | --- | --- |

However, we note that equating the ratio of concentrations in equation (3) with the ratio of rates, as in equation (4), requires equilibrium or at least steady-state conditions (equation (5))

|  | $\frac{d[N-H_{cl}]}{dt}=0$ | (5) |
| --- | --- | --- |

Furthermore, experiments indicate that isotope effects on folding/unfolding equilibra^15,17^ mainly arise from enhanced hydrophobic interactions and increased strength of solvent-solvent H-bonds in D_2_O, while the replacement of amide protons by deuterons does not affect the protein’s stability during exchange experiments.^18^ In additions, studies on D/H amide isotope effects conclude that a deuterated helical H-bond is destabilized by 9 to 22 cal/mol^19,20^ relative to protiated H-bonds. As compared to the stability of an intrahelical amide H-bond in water (≈1 kcal/mol^21^), these stability differences are minor. As a consequence, the folding/unfolding equilibrium constants of deuterated and protiated H-bonds do not differ substantially (K_op,NH_ / K_op,ND_ ≈ 0.98) and we assume for the further analysis:

|  | $K_{op,NH}\approx K_{op,ND}$ | (6) |
| --- | --- | --- |

In order to evaluate the consequences of these assumptions (equations (4) and (6)), we used the thermodynamic cycle (Supplementary Fig. S1**)** that connects the [N-D]/[N-H] equilibria in closed and open states (left and right branches) to the open/close equilibria K_op,NH_ and K_op,ND_ of amide H(D)-bonds (upper and lower branches):

|  | $\frac{\left[ N-D_{cl} \right]}{\left[ N-H_{cl} \right]}=\frac{K_{op,NH}}{K_{op,ND}}\frac{\left[ N-D_{op} \right]}{\left[ N-H_{op} \right]}$ | (7) |
| --- | --- | --- |

In addition, we consequently applied equilibrium assumptions and equated [N-D]/[N-H] ratios with the ratios of the corresponding rate constants for D/H exchange:

|  | $\frac{k(H\to D)}{k(D\to H)}=\frac{K_{op,NH}}{K_{op,ND}} \frac{k_{ch,HDX}}{k_{ch,DHX}}= \frac{k_{exp,HDX}}{k_{exp,DHX}}$ | (8) |
| --- | --- | --- |

Equation (8) implies that the exchange rate constants k_exp_ determined under EX2 conditions (equation (1)) match the rate constants used in equation (4). As a consequence, we recognize that, under the assumption of equal open/close equilibria for protonated and deuterated amides (equation (6)), the fractionation factor calculated from the kinetic isotope effect (equation (4)) reports the isotope preference of the amide-to-water H-bond rather than that of the intrahelical H-bond. The exchange rate constants in the open state, k_ch,DHX_ and k_ch,HDX,_, are equivalent to the chemical exchange rate constants from a hydrated random coil amide (k_rc,DHX_ and k_rc,HDX_) at a given concentration of the exchange catalyst.^8,9^ The concentration of the exchange catalyst [OH^-^] (or [OD^-^]) for base-catalyzed exchange can be determined from the pH, the autoionization constant (K_W_) and the concentration of H_2_O (or D_2_O)^8,9^:

|  | $k_{ch,DHX}\approx k_{rc,DHX}\left[ OH^{-} \right]=k_{rc,DHX}{10}^{pH}K_{W,H_{2}O}[H_{2}O]$ | (9a) |
| --- | --- | --- |
|  | $k_{ch,HDX}\approx k_{rc,HDX}\left[ OD^{-} \right]=k_{rc,HDX}{10}^{pD}K_{W,D_{2}O}[D_{2}O]$ | (9b) |

The fractionation factor Φ is then calculated according to equation (10) under the assumptions of (i) steady-state conditions in the native state (equation (4)), (ii) isotope-independent open/close equilibria (equation (6)), and (iii) base-catalyzed exchange from the open state (equation (9a) and (9b)):

|  | ${\frac{k(H\to D)}{k(D\to H)}}/{\frac{[D_{2}O]}{[H_{2}O]}}=\frac{k_{rc,HDX}{10}^{pD}K_{W,D_{2}O}}{k_{rc,DHX}{10}^{pH}K_{W,H_{2}O}}=\Phi$ | (10) |
| --- | --- | --- |

Using the chemical rate constants k_rc,DHX_ and k_rc,HDX_ for base-catalyzed exchange from a random coil alanine amide and the autoionization constants for H_2_O and D_2_O,^8^ a value of Φ≈0.2 is found. This ratio simply reflects the well-known fact that the chemical rate constant of base‑catalyzed DHX of an unfolded oligo-Ala peptide is five-fold higher than its HDX rate constant (if pH=pD).^22^

Taken together, these arguments challenge the interpretation of amide exchange kinetics using the concept of isotope fractionation. First, it is incorrect to derive the isotope fraction at a backbone amide from the kinetic constants k_exp_, if equilibrium has not been achieved. Second, the formalism described above suggests that the thus calculated Φ values characterize the stability of the amide-to-water, rather than intrahelical, H-bonds. Therefore, a Φ<1 would indicate strong amide-to-water H-bonds in TM-N_._ Indeed, the reported Φ-values in Cao et al.^5^ are, with the exception of T43, V44 and T48, in a narrow range (0.18‑0.42) close to the value Φ≈0.2 predicted by the considerations detailed above. Major differences are likely to arise from the use of detergent micelles in the latter study.

**Supplementary Methods**

**Circular dichroism spectroscopy.** For circular dichroism (CD) spectroscopy, peptides were dissolved in 80% 2,2,2-trifluoroethanol (TFE) with 2 mM NH_4_-acetate, pH 5 at 50 μM. For each sample, 10 accumulated CD spectra from 190-260 nm were obtained using a Jasco J-710 CD spectrometer with a 0.2 data pitch, 1 sec response, bandwidth 2, 100 nm/min scan velocity, 100 mdeg/cm sensitivity, and a path length of 0.1 cm at 20 °C. Mean molar residue ellipticities ([Θ]_mr_) were calculated based on the peptide concentrations which were estimated by UV spectroscopy using the absorbance of the peptide bond at 205 nm with an extinction coefficient e_205_ = 73.600 mol^-1^cm^-1^. This value was determined by calibration with the homologous peptide SNKWGAIIGLMVGGVVIATVIVITLVMLKKK whose concentration was determined using e_280_ = 5600 mol^-1^cm^-1^.

**Deuterium-Hydrogen and Hydrogen-Deuterium Exchange Measurements by Solution NMR**. Dry A26-55 WT (^15^N/^13^C-labeled at positions G29, G33, G37, G38, I41, V44, M51 and L52) from the Core Unit Peptid-Technologien (University of Leipzig, Germany) was dissolved in 500 µL 80% trifluoroethanol-d2 (TFE-d3) and 20 % H_2_O at pH 5.0 adjusted by adding the corresponding amount of NaOH. Peptide concentrations ranged between 50 and 500 µM. NMR spectra were acquired at 27° C on a 600 MHz AVANCE III spectrometer (Bruker BioSpin, Rheinstetten, Germany) equipped with a TXI cryoprobe. ^1^H-^1^H-TOCSY (mixing time of 60 ms), ^1^H-^1^H-NOESY (mixing time of 200 ms), and ^1^H-^13^C-HSQC were acquired for assignment and structural data. Spectra were recorded with 24 scans and 1000 data points in the indirect dimension. The NMR spectra were analyzed using NMRViewJ (One Moon Scientific).

For DHX, peptides were incubated for seven days at 50°C in 80% TFE-d3 and 20% D_2_O. Whether deuteration was completed was checked by NMR spectroscopy. For exchange, peptides were lyophilized and dissolved in 80% TFE-d2, 20% H_2_O. HDX kinetics of the original protonated peptide was recorded in 80% TFE-d3 and 20% D_2_O. Exchange was recorded at three different pH-values (~4, 5, and 6.5) to mimic very short and very long incubation times which expands the coverage of exchangeable protons, using the known correlation of exchange rate and pH value. pH was adjusted using NaOD and DCl. Eleven TOCSY or ClipCOSY^23^ spectra with an experimental time of 3 h 26 min each (mixing time 30 ms, 24 scans, 300 data points in the indirect dimension) were acquired sequentially. For the ^15^N^13^C-labeled WT peptide, eleven additional fast ^1^H-^15^N-HSQC spectra were recorded (2 scans, 128 points in the indirect dimension) in between the TOCSY spectra.

To monitor exchange rates, intensity changes of Hα/HN (TOCSY, 30 ms mixing time) or HN/N (^1^H^15^N-HSQC) cross peaks were monitored. The H to D and D to H exchange rate constants were obtained fitting the cross-peak intensities over time (see Supplementary Fig. S7). For H to D exchanges, the equation for fitting is shown in equation (11).

|  | $y=ae^{-k_{exp,HDX}t}+c$ | (11) |
| --- | --- | --- |

for D to H exchange the fit function is shown in equation (12).

|  | $y=c-ae^{-k_{exp,DHX}t}$ | (12) |
| --- | --- | --- |

where t is time, a and c are constants and k_HD_ and k_DH_ are the apparent rate constants. Rate constants were calculated for all three pH values and then scaled to pH 5.

**Stochastic Sampling of Start Conformations for MD Simulations**

In order to achieve exhaustive sampling of the conformational space, simulations were initiated from varying start conformations. For this purpose, the PyTMD approach was implemented, which in general is a Python3 wrapper around the CHARMM molecular dynamics software package.^24^ PyTMD combines side-chain rotamer combinations from databases with simulated annealing in an implicit membrane environment. Initially, an ideal α-helix in terms of backbone dihedral angles^25^ was assembled from the sequence. To adjust site-chain orientations, rotamer combinations were extracted from a backbone-dependent database^26^ according to their population, and the side-chain dihedral angles were adjusted accordingly. Using this approach, 2500 conformations were sampled, and each was placed in an implicit membrane slap described by the GBSW model.^27^ Model parameters are outlined in Supplementary Tab. S1. For placement, the center of mass of the TMD sequence, as annotated in the UniProt database^28^, was shifted to the origin of the coordinate system, and the helical axis was aligned with the z‑axis.

Before simulated annealing, each conformation was minimized for 100 steps of steepest descent (SD), followed by minimization with the adopted basis Newton-Raphson algorithm (ABNR). Simulated annealing was performed in 25 consecutive steps. Initially, the system was heated for 400 ps from 0 K to 800 K. This was followed by 10 ps dynamics at constant temperature (800 K). Cooling was conducted in 23 steps of 100 ps length, following a stepwise profile as proposed by Kannan & Zacharias (2009)^29^. Temperatures included in the temperature profile were: 800 K, 755 K, 710 K, 670 K, 640 K, 615 K, 590 K, 565 K, 540 K, 520 K, 500 K, 480 K, 460 K, 440 K, 420 K, 400 K, 385 K, 370 K, 355 K, 340 K, 325 K, 310 K, 300 K. During simulated annealing, temperature fluctuations were kept in a range of ±5 K using a Berendsen thermostat. Integration was performed in steps of 2 ps, using the Leapfrog algorithm and SHAKE. Non-bonded neighbor lists were computed up to 20 Å. vdW interactions were threaded up to 16 Å with vdW-switching starting at 15.9 Å.

During the simulated annealing runs, the backbone dihedral angles of the TMD were constrained with force constants of 4.9 (Φ) and 2.2 kcal/mol (Ψ), respectively. The values were derived from the fluctuation windows as measured by NMR^25^.

**Structure Clustering for Determination of Starting Conformations**

In order to obtain a set of representatives from the sampled conformations, all conformations were clustered by the pairwise RMSD between the C_α_ atoms of their TMDs. The pairwise distance matrix $d_{ik}$ was initially computed between all sampled conformations in the dataset.

For simulations in 80 % TFE, the conformations were clustered by affinity propagation clustering ^30^ as implemented in Scikit-learn ^31^. The initial preference was set to the highest similarity $\max\left\{ s_{ik} \right\}$ and the damping factor λ to 0.98. Because affinity propagation works with similarities between data points $s_{ik}$, $d_{ik}$ was transformed by the following formalism:

|  | $s_{ik}= -d_{ik}= -RMSD_{ik}$ | (13) |
| --- | --- | --- |

For the sampled 2500 conformations, this resulted in 78 clusters and related centroids. The centroids were used as starting points for simulations.

Instead of 78 simulations of 200 ns for 80 % TFE, a single 2 µs simulation was conducted for POPC. Hence, a different clustering approach was used, which applied hierarchical clustering with complete linkage. Therefore, a hierarchical tress was built from the pre-computed pairwise distance matrix $d(i,k)$, using the clustering routine as implemented in SciPy ^32^. The number of clusters was determined by the elbow method in the cost-function plot. For each cluster, the centroid was computed from the pairwise distances between structures in the cluster $d_{ij}$:

|  | $Centroid={argmax}_{i}\sum_{j} e^{\frac{-\beta d_{ij}}{\sigma(d_{ij})}}$ | (14) |
| --- | --- | --- |

The centroid of the highest populated cluster was used as initial conformation for the POPC simulations.

**Determination of TMD Orientation in the POPC Membrane.** The orientation of the TMD (G29-L52) in the membrane bilayer was accessed by its tilt (τ) and azimuthal (ρ) rotation angles. τ was computed as the inner product between the membrane normal vector (z-axis) and the TM‑helix axis vector. The helical axis vector was computed by singular value decomposition of the helix axis points determined by a differential geometric approach.^33^ The C_α_ atom of G33 was used as a reference instead of the first residue of the helix.

Insertion depths were calculated as the Z coordinate of Cα atoms, relative to the membrane coordinate system. Therefore, the membrane and the inserted peptide were translated to align the phosphate head’s center of geometry with the origin of the coordinate system.

**ε-Site Accessibility.** In order to assess the accessibility of the ε-site, the rise per residue (RPR) was calculated between the C_α_ atoms of residues T48 and L49 as well as between L49 and V50.^34^ For this purpose, we used a differential geometric approach which fits a cubic spline through three consecutive C_α_ atoms.^35^

**Supplementary Tables**

**Supplementary Table S1.** Settings for the GBSW model used in the PyTMD protocol

| **Parameter** | **Setting** | **Description** |
| --- | --- | --- |
| SW | 0.3 Å | Half smoothing length |
| SGAMMA | 0.03 kcal mol^-1^ Å^-2^ | Nonpolar surface tension coefficients |
| DGP | 1.5 Å | Grid spacing for lookup table |
| TMEMB | 40.0 Å | Thickness of low-dielectric membrane slab |
| MSW | 2.5 Å | Half membrane switching length |
| NANG | 50 | Number of angular integration points |

**Supplementary Table S2.** Numerical values of DHX rate constants and ΔG values derived from ETD-MS

|  | A28-55 WT | | A28-55 I45T | |
| --- | --- | --- | --- | --- |
| Amide | **lg(k_exp,DHX_)[min^-1^]^1^** | **ΔG [kcal/mol]^2^** | **lg(k_exp,DHX_)[min^-1^]^1^** | **ΔG [kcal/mol]^2^** |
| I32 | 0.02 ± 0.03 |  | 0.14 ± 0.07 |  |
| G33 | 0.02 ± 0.08 | 1.00 ± 0.13 | 0.14 ± 0.06 | 0.81 ± 0.10 |
| L34 | -0.05 ± 0.06 | 0.34 ± 0.12 | -0.05 ± 0.03 | 0.34 ± 0.06 |
| M35 | -0.19 ± 0.05 | 0.93 ± 0.09 | -0.25 ± 0.07 | 1.02 ± 0.11 |
| V36 | -0.75 ± 0.08 | 1.23 ± 0.09 | -0.81 ± 0.06 | 1.32 ± 0.09 |
| G37 | -1.02 ± 0.09 | 2.61 ± 0.09 | -0.98 ± 0.10 | 2.56 ± 0.14 |
| G38 | -1.45 ± 0.08 | 3.60 ± 0.09 | -1.45 ± 0.15 | 3.60 ± 0.21 |
| V39 | -1.49 ± 0.11 | 2.35 ± 0.09 | -1.49 ± 0.11 | 2.35 ± 0.15 |
| V40 | -1.87 ± 0.13 | 2.46 ± 0.09 | -1.91 ± 0.22 | 2.51 ± 0.29 |
| I41 | -2.00 ± 0.14 | 2.59 ± 0.09 | -2.42 ± 0.10 | 3.16 ± 0.13 |
| A42 | -2.19 ± 0.11 | 3.69 ± 0.09 | -2.46 ± 0.08 | 4.07 ± 0.10 |
| T43 | -2.11 ± 0.08 | 3.80 ± 0.09 | -2.38 ± 0.09 | 4.17 ± 0.12 |
| V44 | -3.31 ± 0.13 | 4.84 ± 0.09 | -2.98 ± 0.12 | 4.40 ± 0.16 |
| I45 | -3.97 ± 0.10 | 5.24 ± 0.09 | -3.35 ± 0.06 | 5.28 ± 0.08 |
| V46 | -3.97 ± 0.11 | 5.16 ± 0.09 | -3.54 ± 0.31 | 5.15 ± 0.42 |
| I47 | -3.86 ± 0.16 | 5.01 ± 0.09 | -3.63 ± 0.36 | 4.78 ± 0.48 |
| T48 | -3.56 ± 0.26 | 5.45 ± 0.09 | -3.45 ± 0.25 | 5.29 ± 0.33 |
| L49 | -3.40 ± 0.27 | 5.11 ± 0.09 | -3.27 ± 0.22 | 4.95 ± 0.30 |
| V50 | -3.04 ± 0.17 | 3.94 ± 0.09 | -2.57 ± 0.14 | 3.32 ± 0.19 |
| M51 | -2.42 ± 0.19 | 4.11 ± 0.09 | -2.70 ± 0.10 | 4.49 ± 0.14 |

^1^ k_exp,DHX_ derived from exponential fits given in Supplementary Figure 4, means ± SE.

^2^ calculated ΔG values, means with 95% confidence intervals.

**Supplementary Figures**


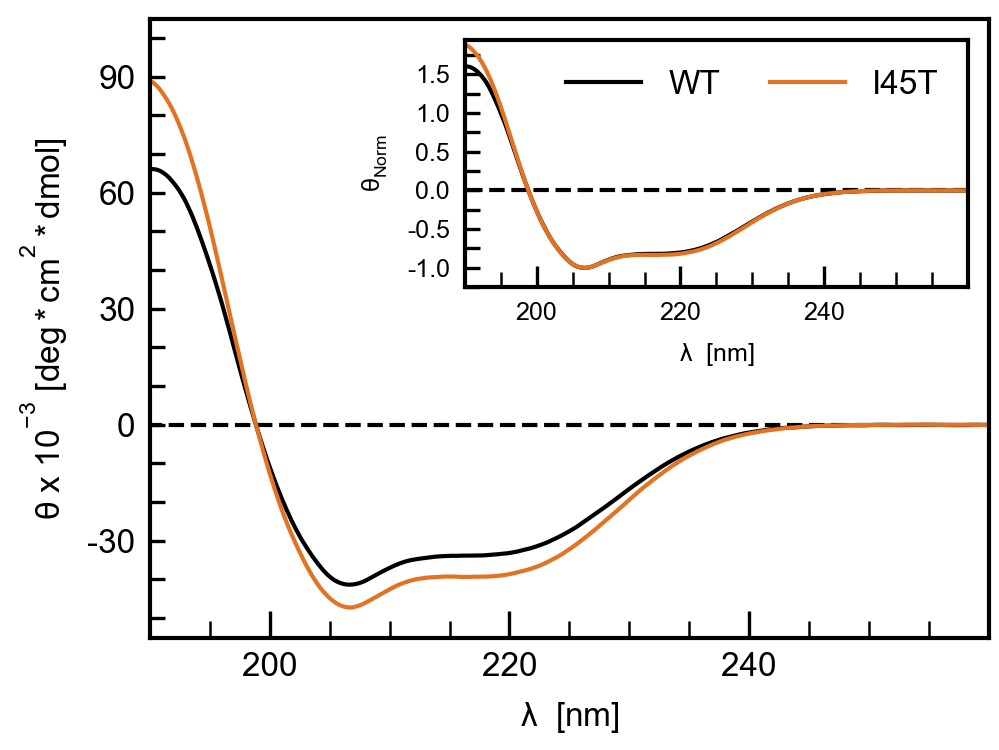


**Supplementary Figure S2.** Circular dichroism (CD) spectra of APP-WT and I45T recorded from 190 nm to 260 nm. The inset shows CD-spectra normalized to the minimal molar ellipticity (θ) at 207 nm (= -1.0). The indistinguishability of the line shapes after normalization indicates that the slight differences of the original spectra reflect uncertainties in the determination of the peptide concentrations, rather than different helicities. Spectra represent averages of 3 replicates.

**
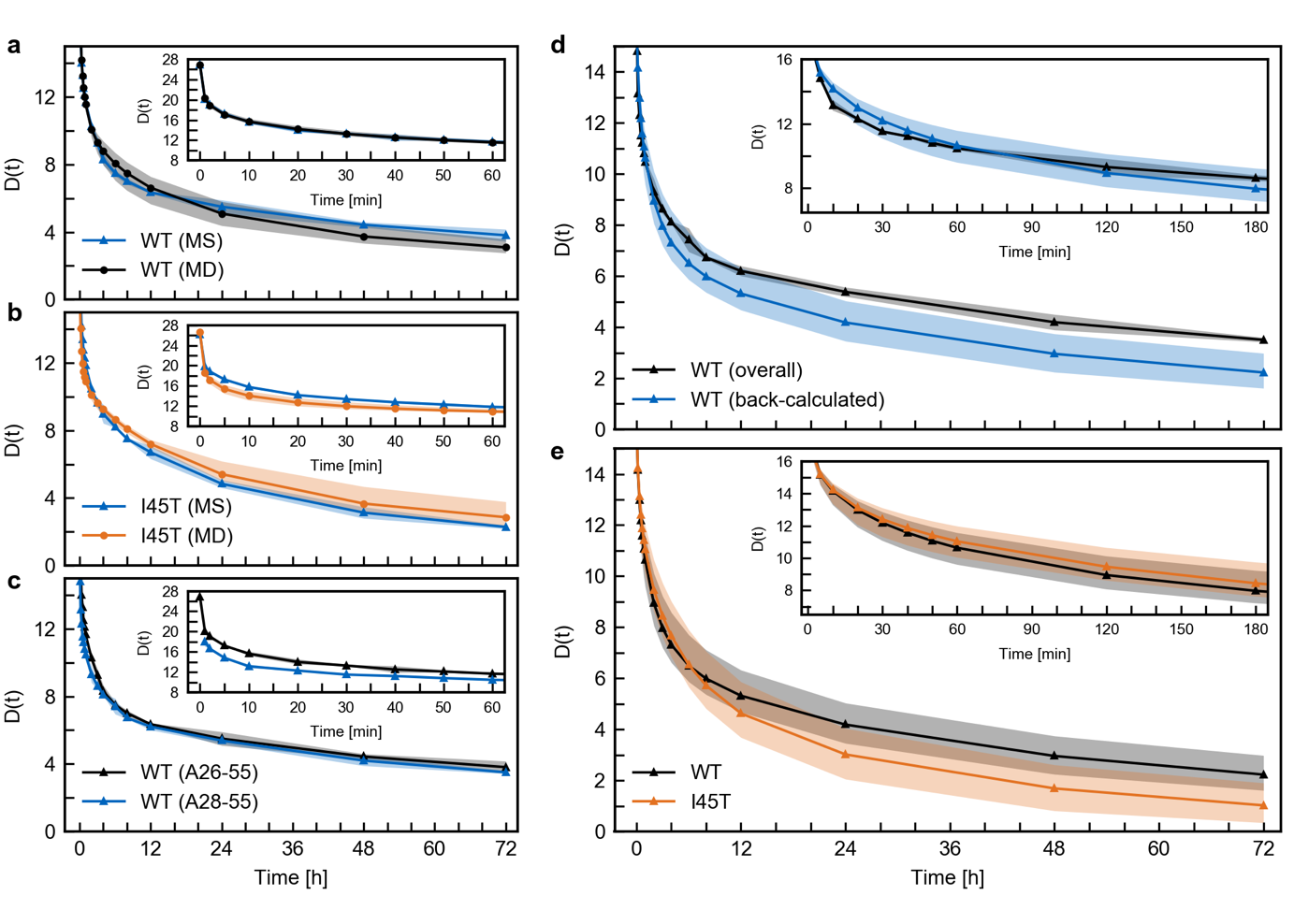
Supplementary Figure S3.** Comparison of overall exchange kinetics. (**a,b**) Comparison between overall exchange measured by MS and calculated exchange from all-atom MD simulations for WT (**a**) and I45T (**b**). Insets show overall exchange during the first 60 minutes. χ^2^ between mean values was 0.4 for WT and 1.652 for I45T. (**c**) Overall DHX kinetics of A26‑55 WT (black line) and A28-55 WT (blue line). For amino acid sequences, see Methods in the main manuscript. (**d**) Comparison between measured overall DHX kinetics of A28-55 WT (black line) and overall DHX kinetics reconstructed from residue-specific k_exp,DHX_ rate constants, as measured by ETD (blue line). (**e**) Comparison of overall DHX of WT (black) and I45T mutant (orange) reconstructed from residue-specific k_exp,DHX_ rate constants, as measured by ETD. Colored areas represent 95 % confidence intervals determined from normal distributions for experiments (n=3) and bootstrap resampling for MD.


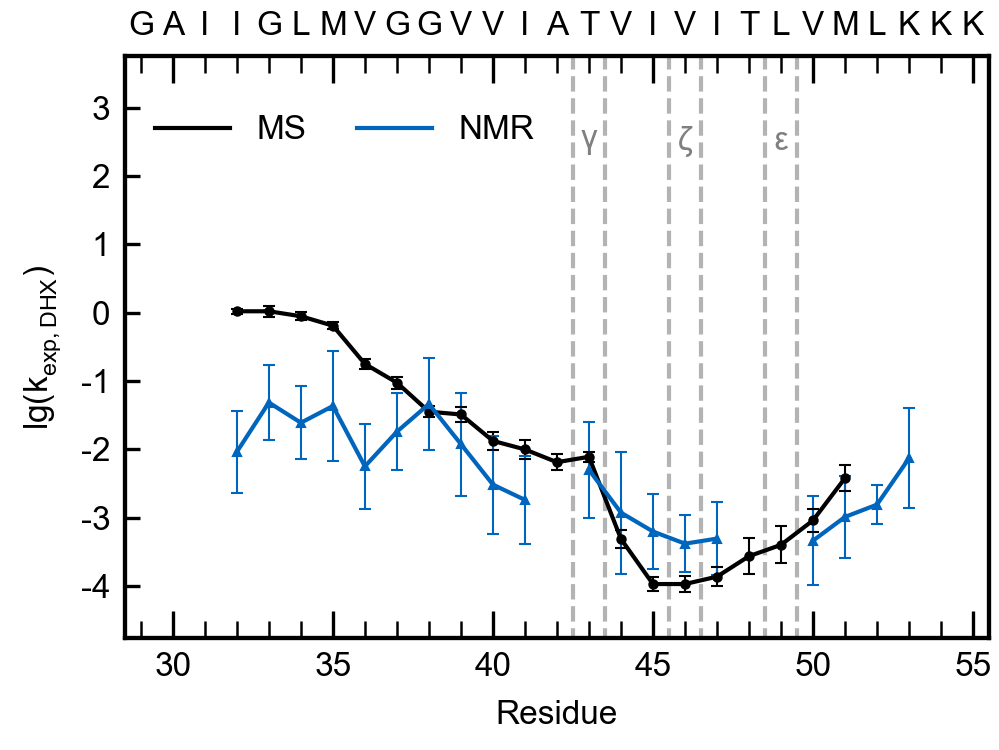


**Supplementary Figure S4.** Comparison of site-resolved DH exchange rates k_exp,DHX_ [1/min], as obtained in 80% TFE by ETD using ESI-TOF mass-spectrometry (MS) or by solution NMR spectroscopy. While both methods indicate faster DHX in TM-N relative to TM-C, discrepancies between both datasets are ascribed to experimental uncertainties and a likely dimerization of the TMD at the high concentrations required for NMR measurements. For ETD‑MS experiments, data points represent mean±SE (n ≥ 3) while for NMR, data points represent mean±var (n = 2).

**
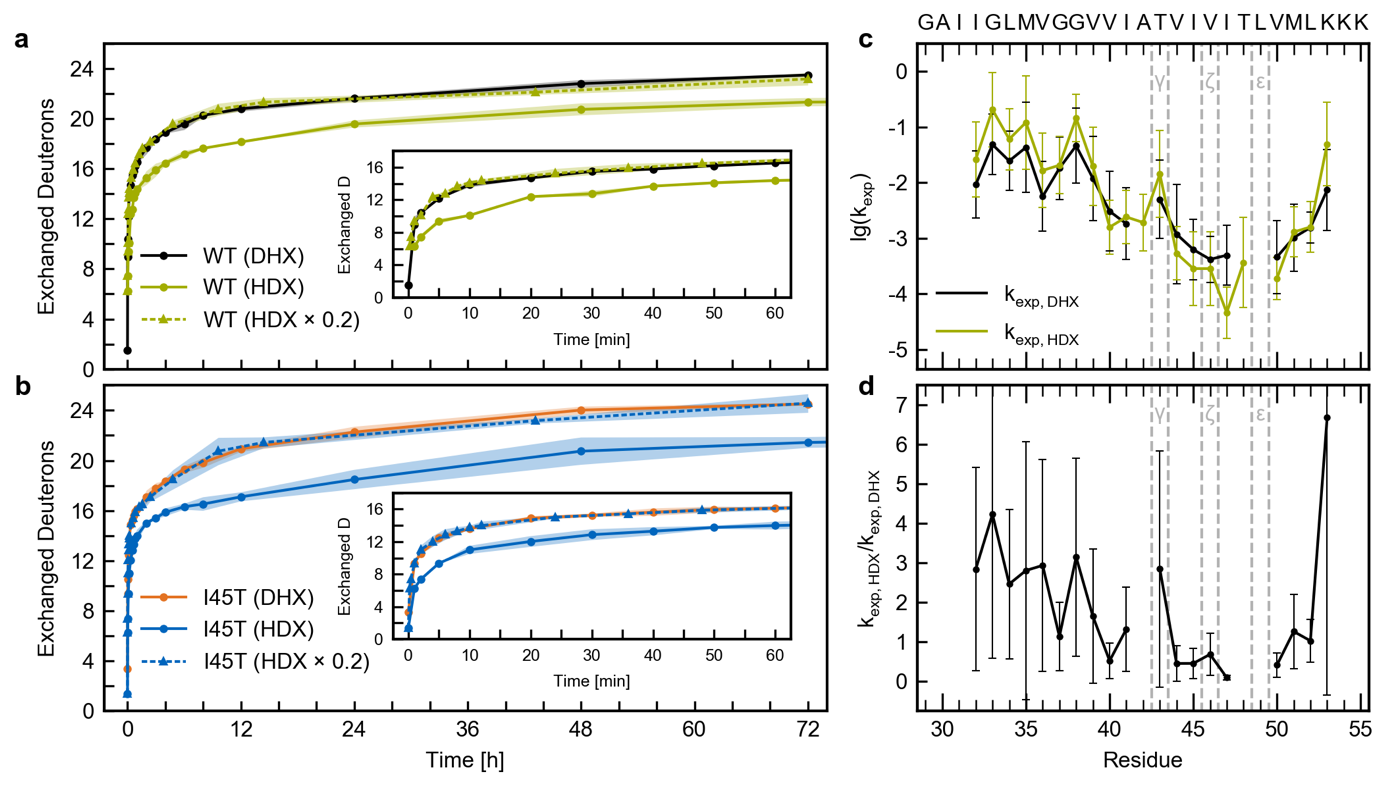
**

**Supplementary Figure S5.** Quantification of differences between k_exp,HDX_ and k_exp,DHX_. (**a,b**) Comparison of overall exchange kinetics measured by MS for DHX and HDX of WT (**a**) and I45T (**b**). For HDX kinetics, the actual measured kinetics are shown, as well as kinetics where the timescale was multiplied by 0.2 (dashed line) to simulate a general 5x higher k_exp,DHX_ for every residue. Note the nearly perfect agreement between DHX kinetics and the 0.2 multiplied HDX kinetics, which confirms our proposed ratio of 0.2 for k_exp,HDX_ / k_exp,DHX_. Insets show the overall exchange during the first 60 minutes (n ≥ 3, mean values ± 95 % CI). (**c**) Site resolved D-to-H (k_exp,DHX_) and H-to-D (k_exp,HDX_) exchange rate constants [1/min] in 80 % TFE by solution NMR (n = 2, mean values ± var) (**d**) Ratio of k_exp,HDX_ to k_exp,DHX_ according to NMR experiments. Note that the residues with the smallest errors show a value of ~0.2.


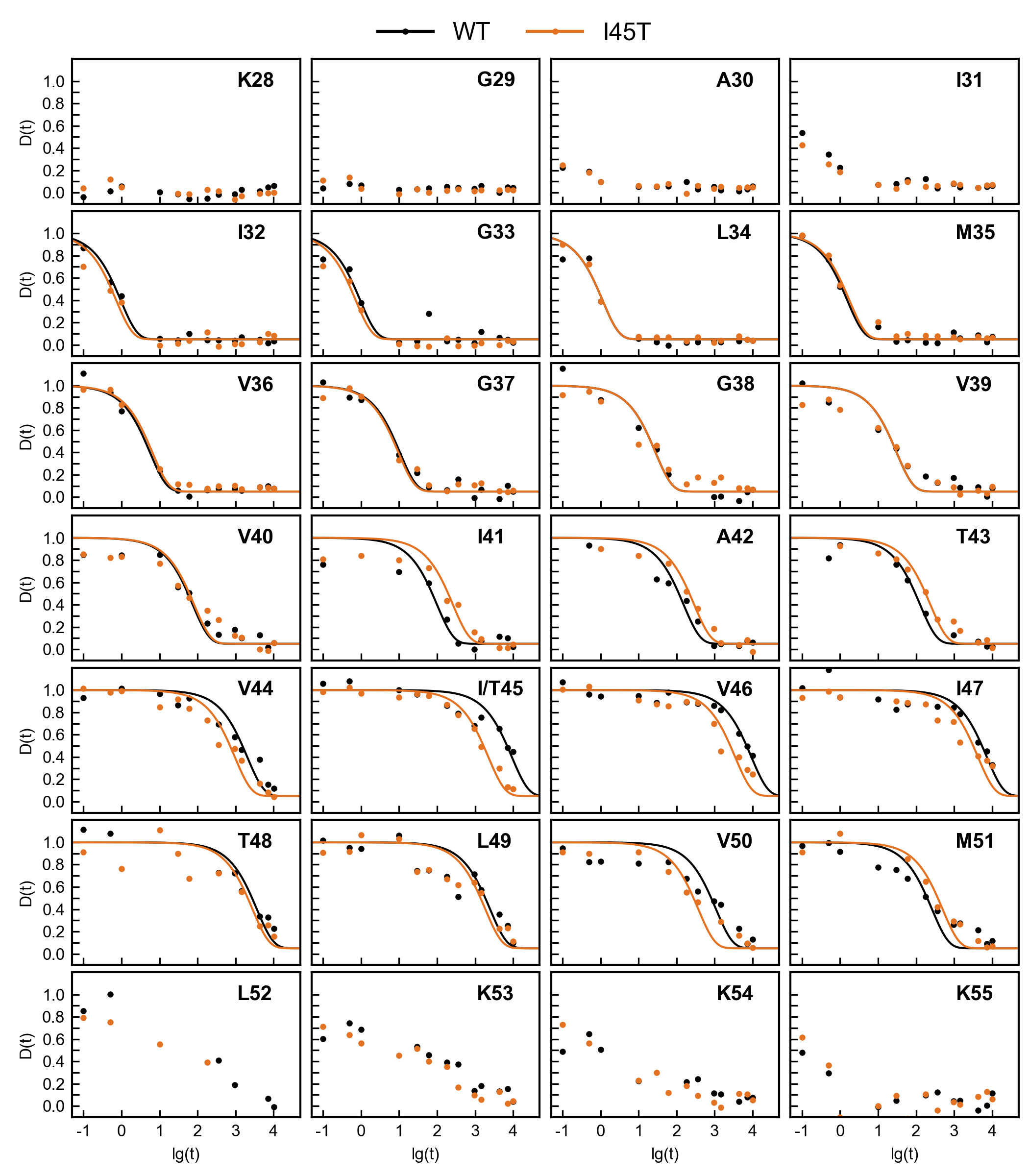


**Supplementary Figure S6.** Residue-specific DHX kinetics obtained from ETD. The calculated deuterium contents D (mean values, n ≥ 3) of the respective amides are plotted against the log of the exchange period t [min]. WT is coded in black, the I45T mutant in orange. Exponential fits are depicted for those residues where kinetics were complete enough to calculate exchange rate constants (given in Fig. 1 of the main text).

**
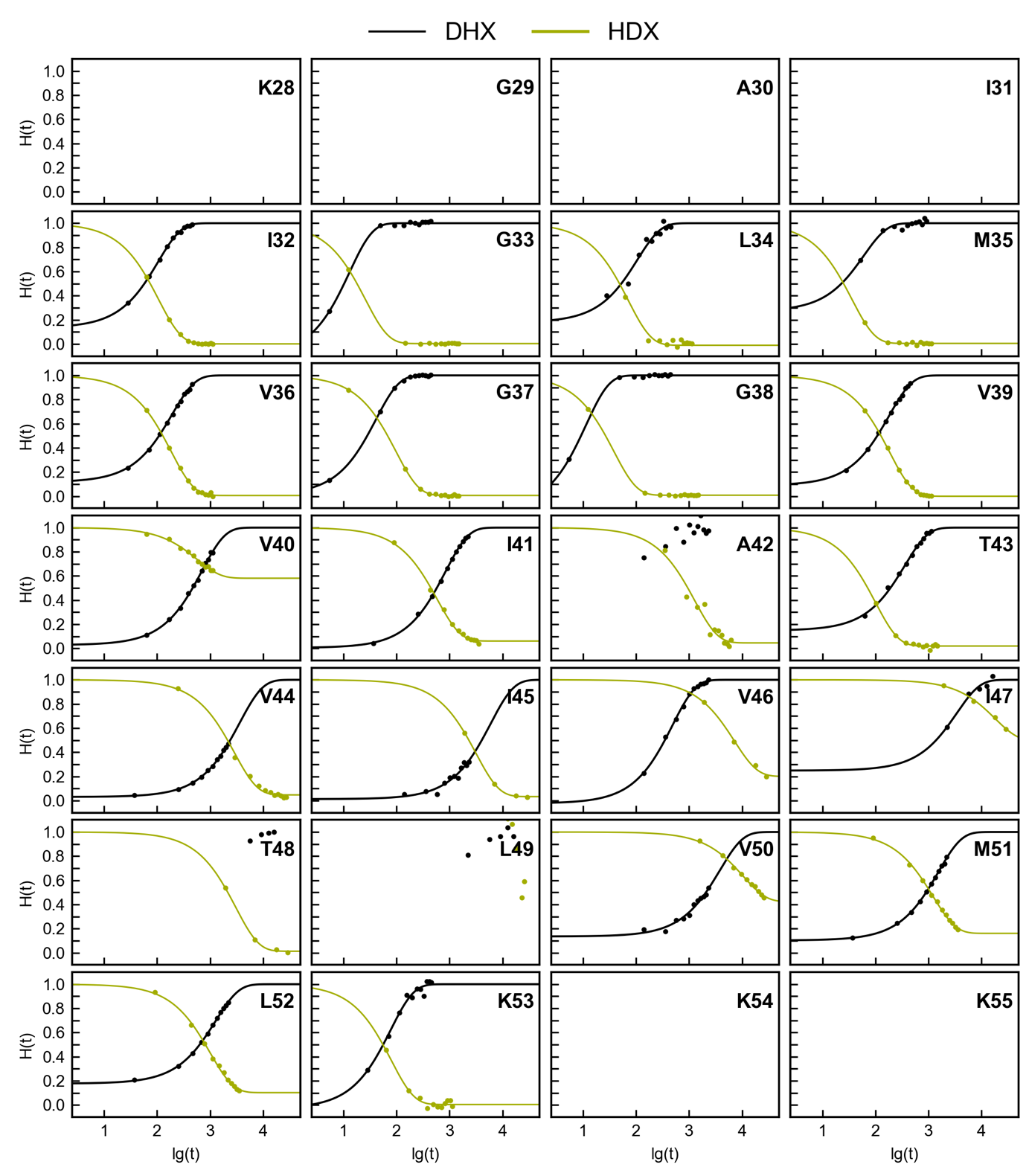
**

**Supplementary Figure S7.** Exemplary residue-specific DHX and HDX kinetics obtained by NMR spectroscopy. The hydrogen contents H(t) of the respective amides are plotted against the log of the exchange period t [min]. Exponential fits are shown by solid lines. Note that in some cases (e.g., M35, A42, I47, T48, L49), the lack of data points at early time points in DHX suggests a high initial H content that is artificial as the deuteration of the peptide at t = 0 was nearly complete.

**
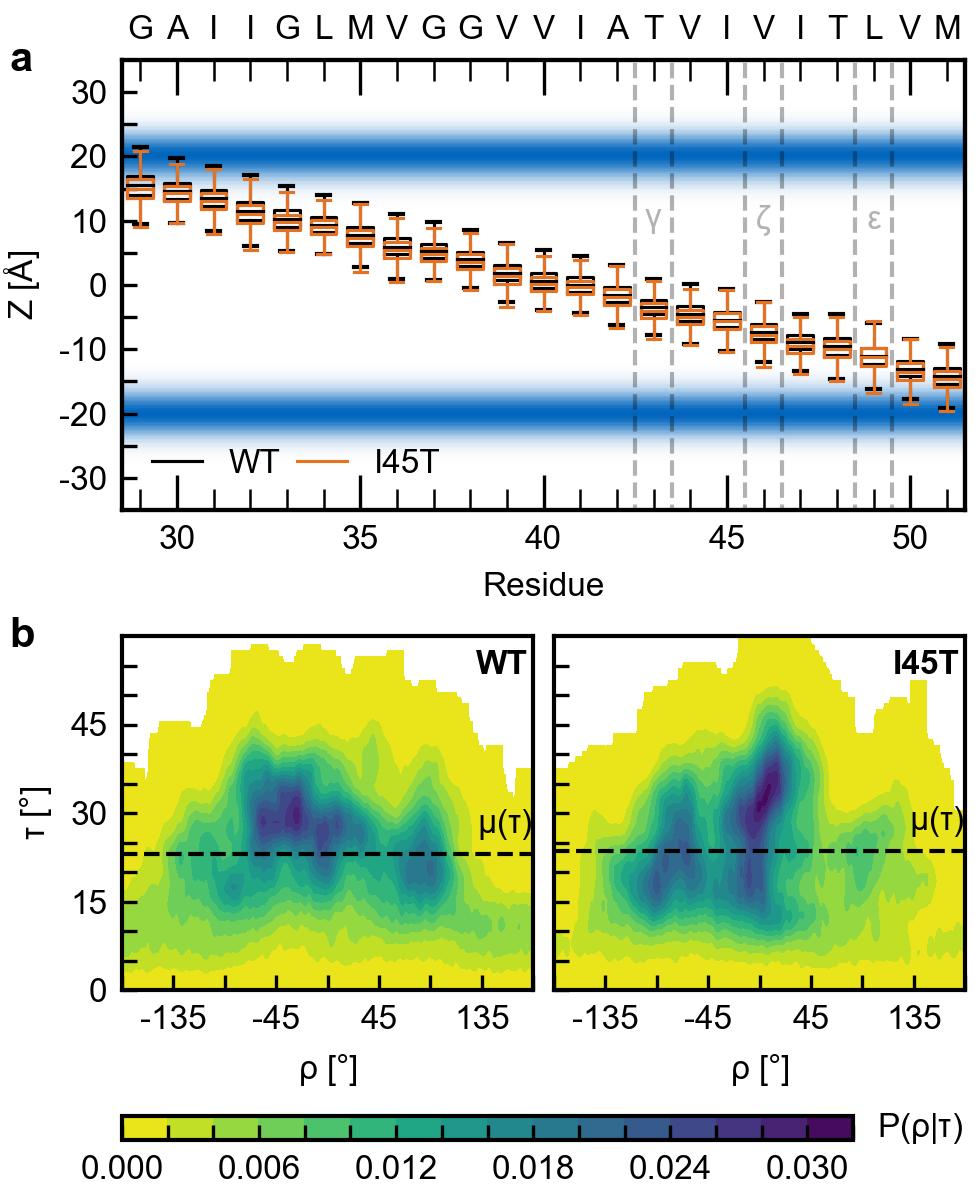
**

**Supplementary Figure S8.** Orientation of the C99 TMD in the POPC bilayer. (**a**) Insertion depths relative to the phosphate heads of the POPC bilayer. Blue areas represent the location of the phosphate heads of the POPC lipids. Insertion depths are shown as boxplots without outliers. (**b**) Probability density of tilt τ and azimuthal ρ rotation angle combinations as filled contour plots. Dashed lines indicate average tilt angles µ(τ). Probability densities are smoothed by 2D Gaussian kernels (σ=2).


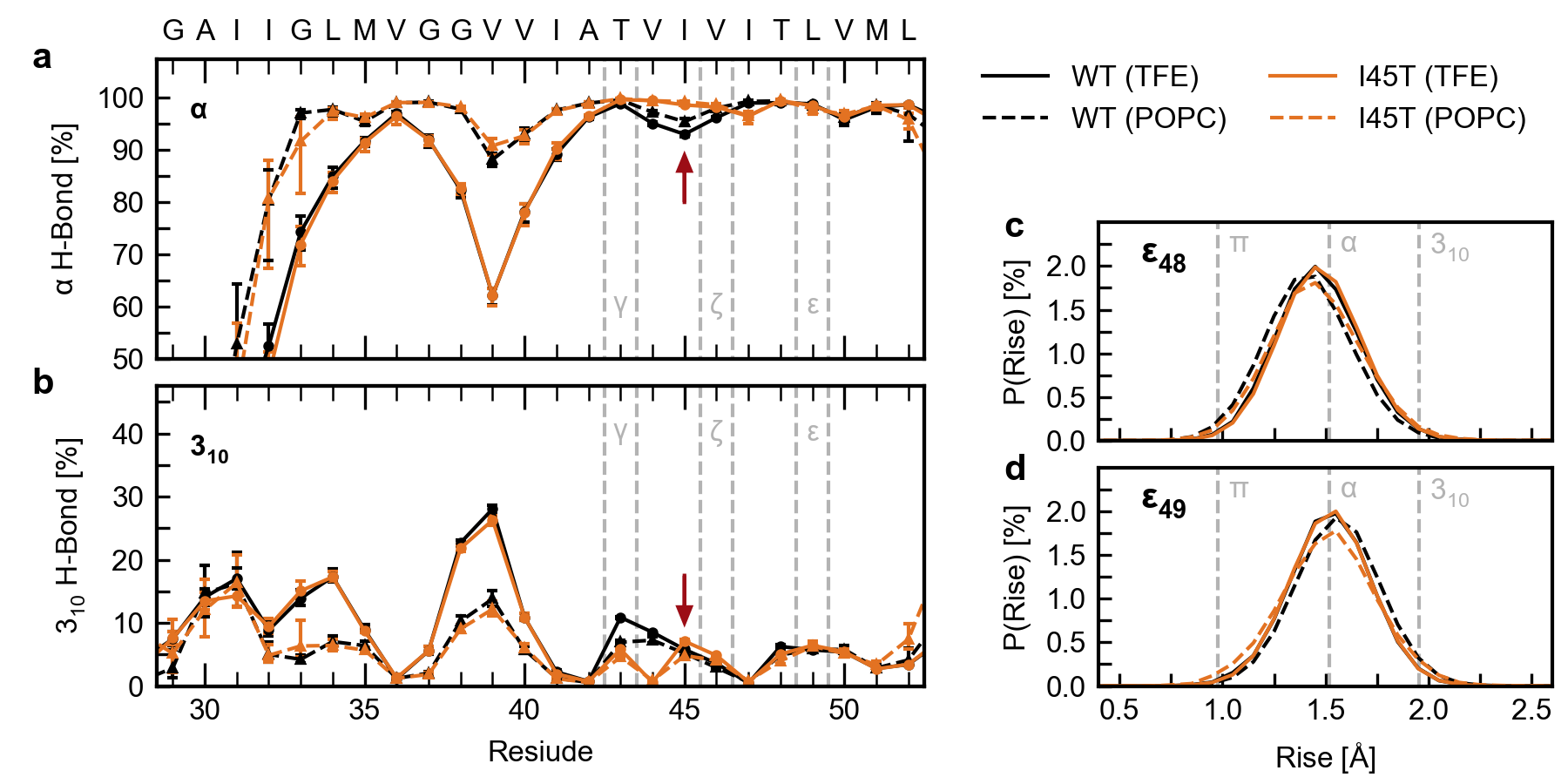


**Supplementary Figure S9.** Local dynamics in 80 % TFE and POPC from MD simulations. (**a**) Occupancies of intramolecular H-bonds between the amide hydrogen at position i and carbonyl oxygen at position i-4 (α helix). (**b**) Occupancies of intramolecular H-bonds between the amide hydrogen at position i and carbonyl oxygen at position i-3 (3_10_ helix). (**c**) Rise between residues T48 and L49, neighboring the ε_48_ cleavage site. (**d**) Rise between residues T49 and V50, neighboring the ε_49_ cleavage site. Red arrows indicate the I45T mutation site. Errors represent 95% confidence intervals from bootstrap resampling.


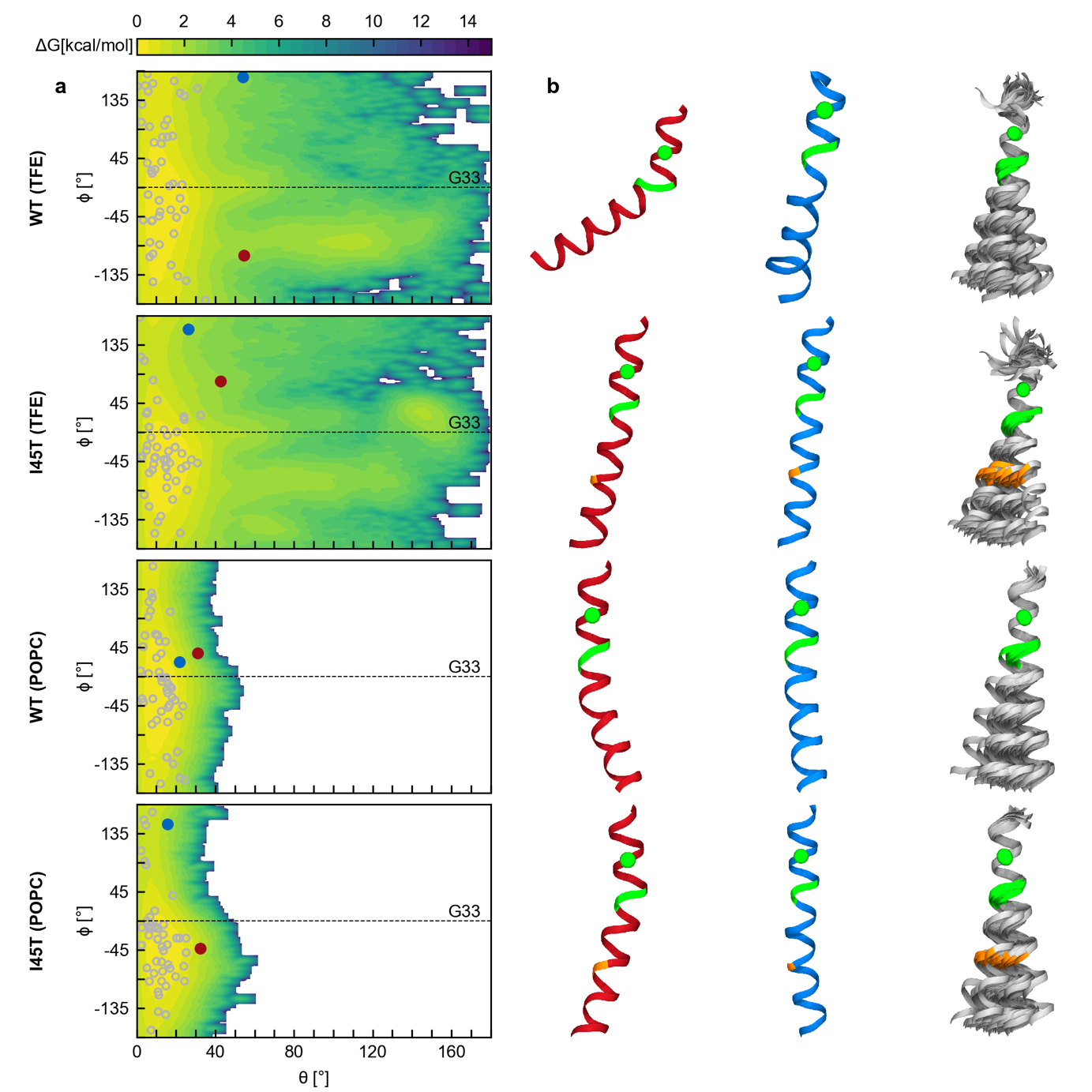


**Supplementary Figure S10.** Free Energy landscape of bend and swivel angles. (**a**) Free energy maps calculated from the probability distributions of bending (θ) and swivel (Φ) angle presented in Fig. 3a. Markers represent conformations obtained by K-Means clustering of θ and Φ angles. The cluster centroid with the highest bending angle is marked in red, blue markers indicate a randomly chosen cluster centroid with lower bending angle. The remaining cluster centroids shown in Fig. 3b are marked in grey. (**b**) Conformations were coloured according to the markers in (a). Residues G37 and G38 are highlighted in green colour. The I45T mutation site is drawn in orange. A green sphere marks the Cα atom of G33.


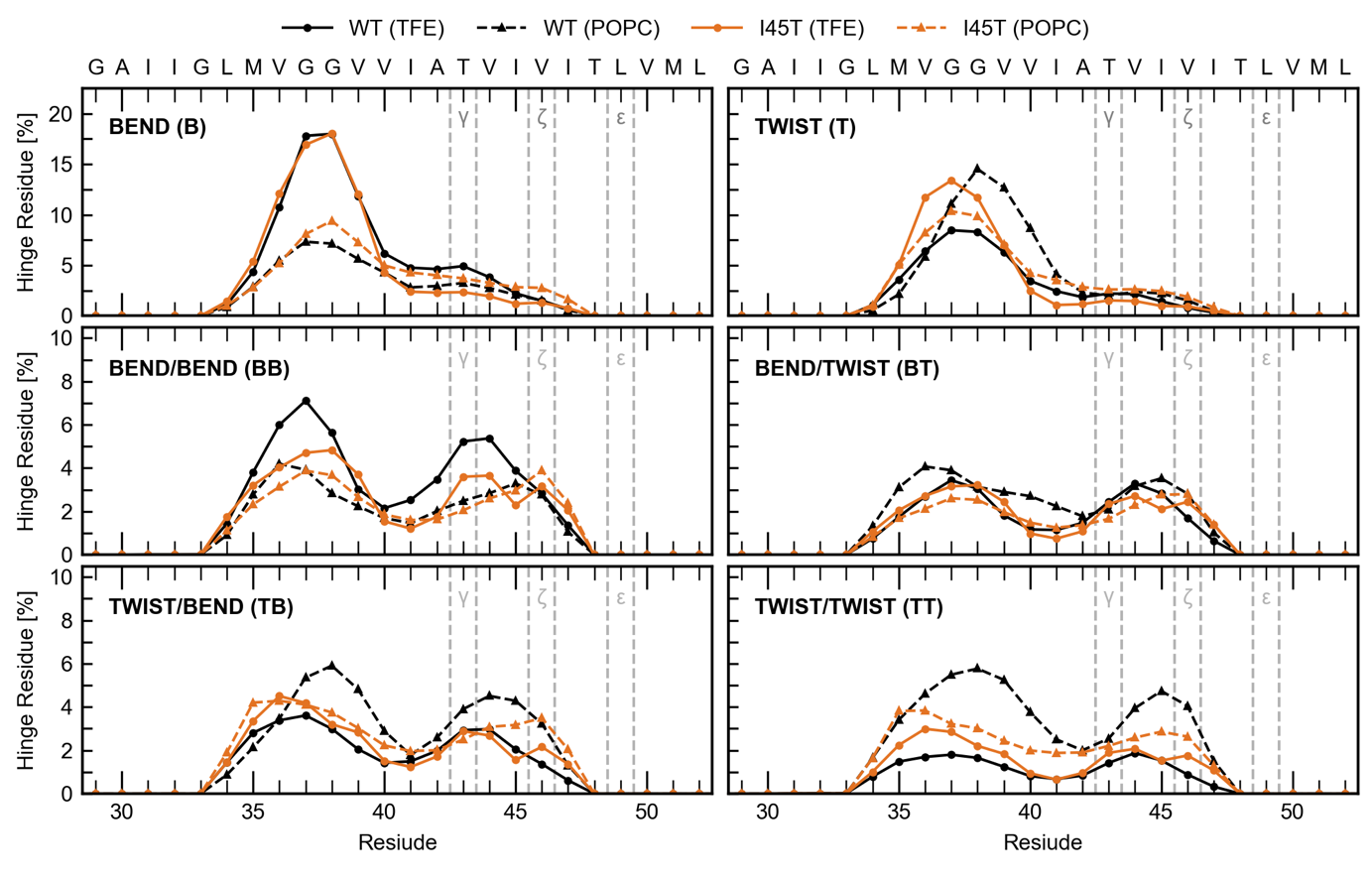
**Supplementary Figure S11.** Probability of an amino acid being classified as part of a hinge by the DynDom program. Hinges of type single B or T coordinate the movement of two quasi‑rigid domains neighboring the hinge. Hinges of type BB, BT, TB and TT coordinate the movement of three quasi-rigid domains by combinations of bending or twisting motions.

**
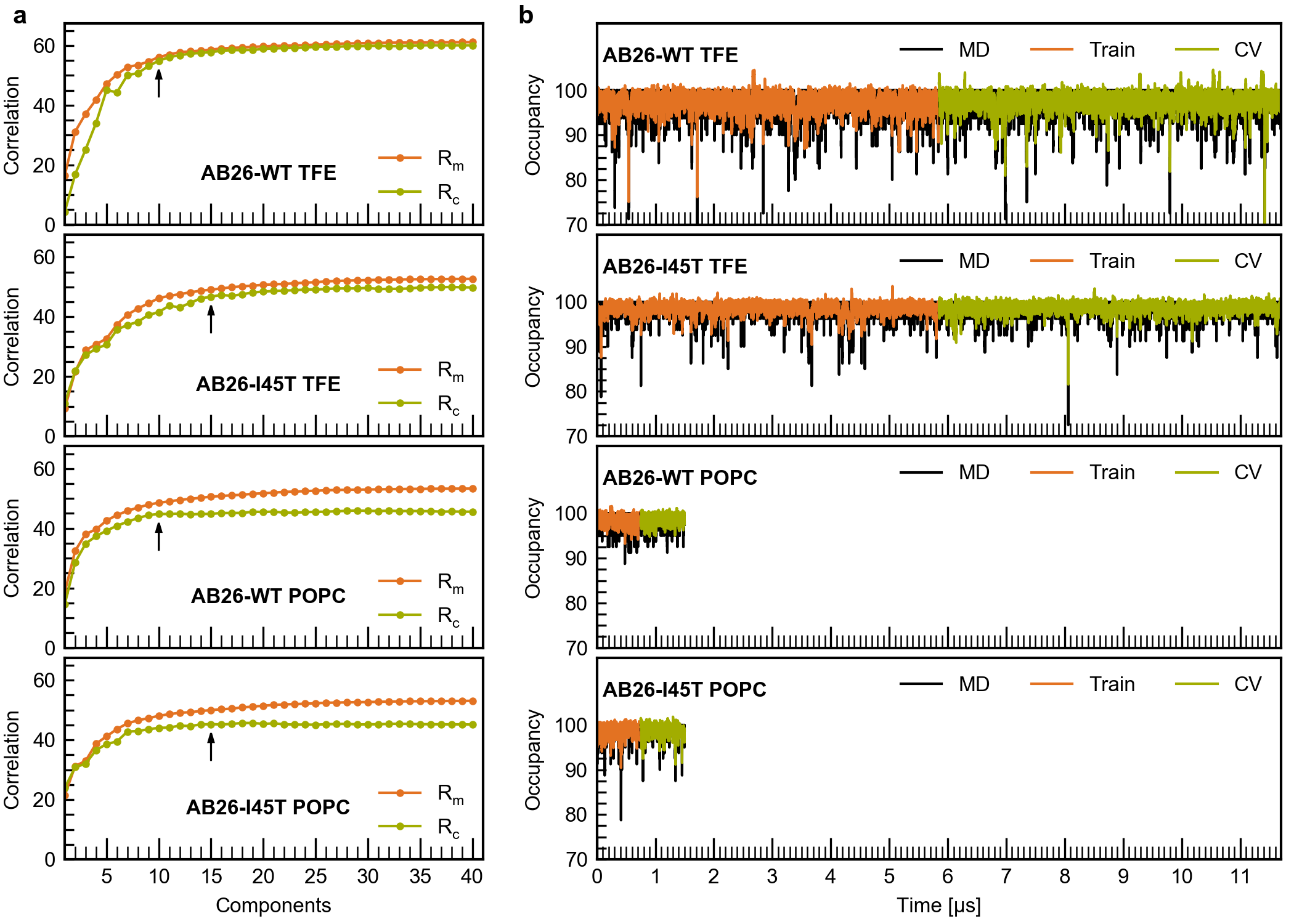
**

**Supplementary Figure S12.** Model building and cross-validation for functional mode analysis. (**a**) Pearson correlation coefficients between data and the model for the training (R_m_) and cross‑validation subset (R_c_) for an increasing number of components. Arrows indicate the selected number of PLS components for the final model. (**b**) Occupancies of backbone H-bonds spanning residues V44 - I47 (black) and predictions from the final PLS model for the training (orange) and cross-validation set (green).

**
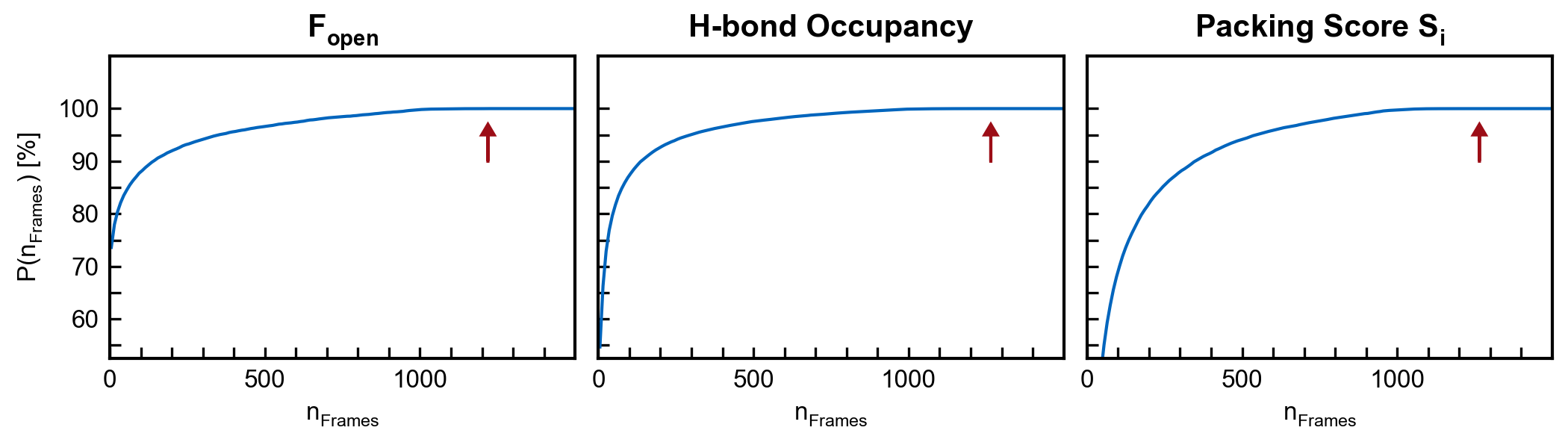
**

**Supplementary Figure S13.** Autocorrelation of first passage times for block averages. Blue lines show the cumulative distribution of first passage times. Red arrows indicate slowest first passage time over all blocks. F_open_ is the fraction of open H-bonds, which is the key determinant in the back calculation of DHX kinetics.

**Supplementary References**

1. Jensen, P. F. & Rand, K. D. in *Hydrogen Exchange Mass Spectrometry of Proteins* (ed. Weis, D. D.) 1–17 (John Wiley & Sons, Ltd, 2016). doi:10.1002/9781118703748.ch1

2. Stelzer, W., Scharnagl, C., Leurs, U., Rand, K. D. & Langosch, D. The Impact of the ‘Austrian’ Mutation of the Amyloid Precursor Protein Transmembrane Helix is Communicated to the Hinge Region. *ChemistrySelect* **1,** 4408–4412 (2016).

3. Loh, S. N. & Markley, J. L. Hydrogen Bonding in Proteins As Studied by Amide Hydrogen D/H Fractionation Factors: Application to Staphylococcal Nuclease. *Biochemistry* (1994). doi:10.1021/bi00170a023

4. Veglia, G., Zeri, A. C., Ma, C. & Opella, S. J. Deuterium/hydrogen exchange factors measured by solution nuclear magnetic resonance spectroscopy as indicators of the structure and topology of membrane proteins. *Biophys. J.* (2002). doi:10.1016/S0006-3495(02)75564-1

5. Cao, Z., Hutchison, J. M., Sanders, C. R. & Bowie, J. U. Backbone Hydrogen Bond Strengths Can Vary Widely in Transmembrane Helices. *J. Am. Chem. Soc.* **139,** 10742–10749 (2017).

6. Pester, O., Götz, A., Multhaup, G., Scharnagl, C. & Langosch, D. The Cleavage Domain of the Amyloid Precursor Protein Transmembrane Helix Does Not Exhibit Above-Average Backbone Dynamics. *ChemBioChem* **14,** 1943–1948 (2013).

7. Skinner, J. J., Lim, W. K., Bédard, S., Black, B. E. & Englander, S. W. Protein dynamics viewed by hydrogen exchange. *Protein Sci.* **21,** 996–1005 (2012).

8. *Protein Folding Handbook*. (Wiley-VCH Verlag GmbH, 2005). doi:10.1002/9783527619498

9. Jensen, P. F. & Rand, K. D. in *Hydrogen Exchange Mass Spectrometry of Proteins* 1–17 (John Wiley & Sons, Ltd, 2016). doi:10.1002/9781118703748.ch1

10. Loh, S. N. & Markley, J. L. Hydrogen Bonding in Proteins As Studied by Amide Hydrogen D/H Fractionation Factors: Application to Staphylococcal Nuclease. *Biochemistry* **33,** 1029–1036 (1994).

11. Khare, D., Alexander, P. & Orban, J. Hydrogen bonding and equilibrium protium-deuterium fractionation factors in the immunoglobulin G binding domain of protein G. *Biochemistry* **38,** 3918–3925 (1999).

12. Edison, A. S., Weinhold, F. & Markley, J. L. Theoretical Studies of Protium/Deuterium Fractionation Factors and Cooperative Hydrogen Bonding in Peptides. *J. Am. Chem. Soc.* **117,** 9619–9624 (1995).

13. Bowers, P. M. & Klevit, R. E. Hydrogen bonding and equilibrium isotope enrichment in histidine-containing proteins. *Nat. Struct. Biol.* **3,** 522–31 (1996).

14. Hvidt, A. & Nielsen, S. O. Hydrogen Exchange in Proteins. *Adv. Protein Chem.* **21,** 287–386 (1966).

15. Krantz, B. A., Moran, L. B., Kentsis, A. & Sosnick, T. R. D/H amide kinetic isotope effects reveal when hydrogen bonds form during protein folding. *Nat. Struct. Biol.* **7,** 62–71 (2000).

16. Bowers, P. M. & Klevit, R. E. Hydrogen Bond Geometry and 2 H/ 1 H Fractionation in Proteins. *J. Am. Chem. Soc.* **122,** 1030–1033 (2000).

17. Makhatadze, G. I., Clore, G. M. & Gronenborn, A. M. Solvent isotope effect and protein stability. *Nat. Struct. Biol.* **2,** 852–855 (1995).

18. Parker, M. J. & Clarke, A. R. Amide backbone and water-related H/D isotope effects on the dynamics of a protein folding reaction. *Biochemistry* **36,** 5786–5794 (1997).

19. Krantz, B. A. *et al.* Understanding protein hydrogen bond formation with kinetic H/D amide isotope effects. *Nat. Struct. Biol.* **9,** 458–463 (2002).

20. Jaravine, V. A., Cordier, F. & Grzesiek, S. Quantification of H/D Isotope Effects on Protein Hydrogen-bonds by h3 J NC and 1 J NC′ Couplings and Peptide Group 15 N and 13 C′ Chemical Shifts. *J. Biomol. NMR* **29,** 309–318 (2004).

21. Bowie, J. U. Membrane protein folding: How important are hydrogen bonds? *Curr. Opin. Struct. Biol.* **21,** 42–49 (2011).

22. Teilum, K., Kragelund, B. B. & Poulsen, F. M. in *Protein Folding Handbook* 634–672 (Wiley-VCH Verlag GmbH). doi:10.1002/9783527619498.ch18

23. Koos, M. R. M., Kummerlöwe, G., Kaltschnee, L., Thiele, C. M. & Luy, B. CLIP-COSY: A Clean In-Phase Experiment for the Rapid Acquisition of COSY-type Correlations. *Angew. Chemie - Int. Ed.* **55,** 7655–7659 (2016).

24. Brooks, B. R. *et al.* CHARMM: The biomolecular simulation program. *J. Comput. Chem.* **30,** 1545–1614 (2009).

25. Page, R. C., Kim, S. & Cross, T. A. Transmembrane Helix Uniformity Examined by Spectral Mapping of Torsion Angles. *Structure* **16,** 787–797 (2008).

26. Shapovalov, M. V. & Dunbrack, R. L. A smoothed backbone-dependent rotamer library for proteins derived from adaptive kernel density estimates and regressions. *Structure* **19,** 844–58 (2011).

27. Chen, J., Im, W. & Brooks, C. L. Balancing solvation and intramolecular interactions: toward a consistent generalized Born force field. *J. Am. Chem. Soc.* **128,** 3728–36 (2006).

28. Bateman, A. *et al.* UniProt: The universal protein knowledgebase. *Nucleic Acids Res.* **45,** D158–D169 (2017).

29. Kannan, S. & Zacharias, M. Simulated annealing coupled replica exchange molecular dynamics--an efficient conformational sampling method. *J. Struct. Biol.* **166,** 288–94 (2009).

30. Frey, B. J. & Dueck, D. Clustering by passing messages between data points. *Science* **315,** 972–976 (2007).

31. Pedregosa, F. *et al.* Scikit-learn: Machine learning in Python. *J. Mach. Learn. Res.* **12,** 2825–2830 (2011).

32. Oliphant, T. E. SciPy: Open source scientific tools for Python. *Comput. Sci. Eng.* **9,** 10–20 (2007).

33. Strandberg, E., Esteban-Martín, S., Salgado, J. & Ulrich, A. S. Orientation and dynamics of peptides in membranes calculated from 2H-NMR data. *Biophys. J.* **96,** 3223–3232 (2009).

34. Götz, A. & Scharnagl, C. Dissecting conformational changes in APP’s transmembrane domain linked to ε-efficiency in familial Alzheimer’s disease. *PLoS One* **13,** e0200077 (2018).

35. Guo, Z., Kraka, E. & Cremer, D. Description of local and global shape properties of protein helices. *J. Mol. Model.* **19,** 2901–11 (2013).
